# The role of pharmacists in the renal multidisciplinary team at a tertiary hospital in South Africa: Strategies to increase participation of pharmacists

**DOI:** 10.4102/hsag.v25i0.1357

**Published:** 2020-08-26

**Authors:** Tebogo L. Manyama, Rendani M. Tshitake, Noko B. Moloto

**Affiliations:** 1 Department of Pharmacy, Faculty of Health Sciences, University of Limpopo, Mankweng, South Africa

**Keywords:** pharmacists, chronic kidney disease, multidisciplinary team, strategies, scope of practice, barrier

## Abstract

**Background:**

Pharmacists are often marginalised from participating fully in a Multidisciplinary Team (MDT). Pharmacists can contribute in the renal MDT by minimising drug-related problems and optimising therapy.

**Aim:**

The study aimed to explore the current role of pharmacists in renal care at a tertiary hospital in South Africa, and to recommend strategies to improve their participation in the renal MDT.

**Method:**

An exploratory descriptive qualitative study was conducted using semi-structured interviews. The participants were selected using purposive sampling. The audiotaped interviews were transcribed exactly as spoken and analysed using thematic content analysis.

**Results:**

Three themes emerged from the analysis: pharmacist’s current scope of practice within the renal MDT, potential future roles of pharmacists, and perceived barriers to participation of pharmacists within the renal MDT. Furthermore, participants provided recommendations to increase pharmacist’s participation in the renal MDT: that is standardisation of practice, skills development of both pharmacist and pharmacist assistants and recognition of pharmacist services in the wards.

**Conclusion:**

The role of pharmacists at Pietersburg Hospital is the official name of the hospital is confined to stock management and dispensing. Efforts should be made to improve the participation of pharmacists in the MDTs with the intention to standardise the practice of pharmacists in the wards, equip both pharmacists and pharmacist assistants with the necessary skills and recognise pharmacist’s services in the wards.

## Introduction

Chronic kidney disease (CKD) is a growing health concern and a major cause of morbidity and mortality worldwide (Neuen et al. 2017; Stevens, Viswanathan & Weiner 2010). Lack of awareness, failure to recognise early signs, and improper management of CKD contribute to CKD’s burden (Raymond et al. 2010). Patients with CKD and those with severe forms of CKD are at an increased risk of being exposed to drug-related problems, including adverse drug reactions and drug–drug interactions. The complex nature of the therapy, altered pharmacokinetics of the drugs and reduced renal function further dispose patients to drug-related problems (Stemer & Lemmens-Gruber 2011).

The renal Multidisciplinary Team (MDT) integrates the interventions of nephrologists, physicians, nurses, dieticians and pharmacists who provide care to patients with renal disorders (Chen et al. 2012). When properly implemented, this team meets the health demands of the patients when compared to individual patient–physician care (Clarke 2013). The role of a pharmacist in the renal MDT includes identifying, addressing and preventing drug-related problems, providing recommendations on drug selection and treatment plans, adjusting doses, preventing use of contraindicated drugs and enhancing patient adherence (Biradar et al. 2012; Splawski & Minger 2016). The inclusion of a pharmacist in the renal MDT further improves quality of life, reduces unnecessary drug use, and hospitalisation, and improves outcomes for anaemia, hypertension and diabetes mellitus for patients with CKD (Mekonnen et al. 2013; Odum & Whaley-Connell 2012). Pharmacists are also expected to play a role in renal drug cost management. These aspects contribute in cost saving for both patients and institutions and would be essential in developing countries struggling with resource limitations. In resource-limited countries with human resource challenges, pharmacists can also bridge the gap between physicians and patients by providing direct patient care (Shrestha, Shrestha & Palaian 2019). Renal therapy is costly and complex (Aviles-Gomez et al. 2006), and therefore pharmacists have the potential to contribute to the renal MDT (Al Raiisi et al. 2019). To further highlight the essential role of a pharmacist, Shulman et al. (2015) observed that one in six prescriptions requires an intervention from the clinical pharmacist.

Despite numerous studies demonstrating the positive outcomes of pharmacists practicing in wards (Chisholm-Burns et al. 2010; Chen et al. 2012; Jacobi 2016; Stemer & Lemmens-Gruber 2011), the role of pharmacists in the renal MDT remains limited especially in developing countries (Bronkhorst et al. 2014; Gray, Riddin & Jugathpal 2016; Mekonnen et al. 2013; Salgado et al. 2014). Factors such as conflicting departmental priorities, workload, resource-related constraints (space, finance and qualified personnel), system-related constraints, lack of clinical knowledge and discrepant attitudes of pharmacists, doctors and nurses are some of the barriers to pharmaceutical care (Acheampong & Anto 2015; Walker et al. 2014). It is against this background that the study aims to explore the role of pharmacists in renal care and proposes strategies based on their participation in the renal multidisciplinary health care team at a tertiary hospital in Limpopo province, South Africa.

## Methods

### Design and study participants

An exploratory qualitative research method was conducted among members of renal MDT and pharmacists at a tertiary hospital in South Africa. The participants were purposively selected to deliberately select participants with the necessary experiences. Data saturation was achieved with 17 participants. The participants were registered health care professional within the renal MDT as well as pharmacists employed at the hospital.

Data were collected via in-depth, face-to-face semi-structured interviews using an interview guide that was developed by the researcher based on the objectives of the study (Table 1) and verified by experienced qualitative researchers. The interview guide consisted of central questions which were further followed up by probing questions.

**TABLE 1:** Semi-structured interview guide: This table presents the central questions that were followed during the interview process.

1. Briefly explain the current roles of a pharmacist in the renal multidisciplinary team and renal unit?
2. Based on the needs of the renal unit, what more roles can the pharmacists undertake within the renal multidisciplinary team to help in the management of patients with CKD?
3. What do you think could be the limiting barriers to their participation in the management of patients and within the renal MDT?
4. What suggestions or strategies do you think should be implemented to increase the participation of pharmacist in the renal multidisciplinary team?

MDT, multidisciplinary team; CKD, Chronic kidney disease.

### Data analysis

The interviews with the participants were audio-recorded and transcribed exactly as spoken. Thematic content analysis was used to analyse the interviews’ transcripts. Following this method of analysis, the researcher read the transcripts repeatedly, identified emerging topics and categorised the topics as themes and subthemes. Analysis was conducted by the researcher and an independent coder, thereafter results were reconciled for differences and agreement.

### Trustworthiness

In this study, trustworthiness was achieved through measures of credibility, transferability, dependability and conformability. In order to achieve credibility, the researcher spends time at the hospital providing voluntary service. In addition, the researcher obtained the perceptions of experienced qualitative researchers during the research process and conclusion. Transferability was achieved through purposive sampling, ensuring that the research focuses on key participants who are knowledgeable of the issues under this study and the use of open-ended questions during the interview process. An independent coder was used to enhance dependability and conformability. After transcription, a copy of the transcript was given to an independent coder who was well-versed in qualitative data-analysis in order to conduct a different data analysis from the researchers and the findings were compared for similarities or differences and consolidated after reaching consensus.

### Ethical consideration

A written consent was obtained from participants who were assured of the confidentiality and anonymity of their responses. Ethical approval was obtained from the Turfloop Research Ethics Committee (Ref: TREC/262/2017).

## Results

A total of 17 health care professionals participated in this study, of whom 14 (82.4%) were females and three (17.6%) were males. Pharmacists were the majority with nine participants and the remaining eight (47.1%) participants were other healthcare professionals practicing in the renal unit. The renal MDT was comprised of a nephrologist, physician, two dieticians, a transplant coordinator, a unit manager, and two professional nurses. Participants’ age ranged between 29–57 the age group. All the participants had a working experience of over 5 years in practice.

Three themes emerged from the interviews. The main themes and subthemes are illustrated in Table 2. Respondents from the renal multidisciplinary team (MDT) are denoted by using the symbol Rtm (renal team), while P symbolises pharmacists.

**TABLE 2:** The identified themes and sub-themes from the interview transcripts.

Themes	Sub-themes
Pharmacist’s current scope of practice with the renal multidisciplinary team	Stock management Lack of clinical participation in the renal MDT
Potential future role of pharmacists	Promote rational drug use Patient education/counselling Monitoring of patients
Barriers to participation of pharmacists in the renal MDT.	Lack of human resource Attitude of pharmacists Attitude of other health care providers Pharmacists’ education and training Lack of recognition of pharmacy services

MDT, multidisciplinary team.

### Theme 1: Pharmacist’s current scope of practice within the renal multidisciplinary team

The majority of the renal MDT members spontaneously reported to be interacting with pharmacists in the unit during stock management, which was also confirmed by the majority of pharmacists:

‘The pharmacists in the unit are responsible for assisting in stock ordering and when we need urgent supplies.’ (Rtm 10)

and:

‘ensures stock availability.’ (P 9)

Seemingly, pharmacists were only responsible for the ordering and supply of medicines to the renal unit. It was evident from the interview that pharmacist seldom participate in the day-to-day management of patients in the renal unit. This was also supported by this statement:

‘[*W*]e send pharmacists to wards but not necessarily to deal with the patientsc[…].’ (P 6)

*‘*Unfortunately, at present, our intervention as pharmacists is just from the pharmacy department and from the files we get.’ (P 8)

### Theme 2: Potential future roles of pharmacists in the renal multidisciplinary team

While some of the participants from the renal unit seemed to be content with the current role of the pharmacists, the majority of the participants indicated that they could play a major in the clinical care of renal patients. Participants from the renal MDT and pharmacists mentioned that reviewing of the prescription was the fundamental role pharmacists should carry out:

‘Pharmacists can play a vital role in reviewing of medication that is already prescribed.’ (Rtm 10)

and to:

‘[…] reduce the unnecessary treatment that the patients are taking.’ (P 5)

Participants reported that pharmacists should oversee dosage adjustments and prevent the use of contraindicated drugs:

‘We need a pharmacist’s advice on giving proper dosages […].’ (Rtm 2)

and:

‘[…] to make sure that the medications patients are taking are not actually contraindicated or interacting with other medications.’ (P 14)

Alongside prescription reviews, pharmacists and members of the renal MDT stated that pharmacists should be assisting at the prescribing stage with physicians:

‘Pharmacists can work hand in hand with doctors when prescribing.’ (Rtm 16)

and:

‘[…] we can closely work with the doctor in developing the pharmaceutical care of the patients.’ (P 7)

Patient education was also mentioned by several participants as an integral activity to be conducted by pharmacists in the renal unit:

‘[*P*]harmacists can make sure that our patients understand the medication […].’ (Rtm 10)

and:

‘[*C*]ounsel them especially for example, about the side effects of the drugs.’ (P 14)

‘[…] drug interactions.’ (P 5)

and:

‘[…] on fluid limitations.’ (P 7)

‘I think they will contribute as to how best they can adhere to treatment […].’ (Rtm 1)

### Theme 3: Barriers to participation of pharmacists in the renal multidisciplinary team

Through the interviews, several barriers that would hinder pharmacists’ participation in the renal MDT were identified.

#### Lack of human resource

Shortage of pharmacy staff stood out as a major barrier to the participation of pharmacists during renal care:

‘I think the main issue here is shortage of personnel.’ (Rtm 2)

and:

*‘*[…] the minute one pharmacist is out; a gap is opened in the pharmacy.’ (P 5)

#### Attitude of pharmacists

Some pharmacists reported that attitude of pharmacists was a barrier and clinical roles were regarded as nurses’ roles:

‘I think there is an attitude with or from pharmacists…. they just come in from universities and limit their role to the pharmacy as if they have reached their goal.’ (P 14)

‘[*I*]t [*is*] more of the sister’s duty to do the clinical part and we just look at the medicine side.’ (P 3)

#### Attitude of the health care providers

It was reported that other health care professionals are not accommodating and would prefer to work without a pharmacist:

‘Other health care workers are also not trained to acknowledge or to accommodate pharmacist role inside the ward.’ (P 5)

‘[*S*]ometimes the doctors feel like you want to take their job when you ask too much about what they are doing.’ (P 11)

‘From a pharmacist point of view for them to come and like work here, I don’t know if it will be possible or feasible.’ (Rtm 17)

‘[…] we are fine working without them at the moment. The way we are working at present, I am not complaining.’ (Rtm 16)

#### Pharmacists’ education and training

Lack of clinical skills by pharmacists appeared as one of the barriers:

‘The training we got made us generalists, it doesn’t make us specialists in any form.’ (P 9)

‘I don’t think pharmacists are skilled enough to take part in nursing renal failure patients.’ (Rtm 2)

‘[…] most of us are not doing post graduate standards to continue with professional development which could help us.’ (P 9)

#### Lack of recognition of pharmacy services

Pharmacists consistently reported a lack of recognition of the pharmacy services by other healthcare professionals, hospital management and the South African Pharmacy Council:

‘If a doctor can know this are the benefits I will get from a pharmacist or a nurse instead of just using them as if they are their assistants, for example, […].’ (P 5)

‘I don’t know if it’s an issue of awareness, whether a pharmacist is recognised as part of a team player in the health care context of South Africa and in dealing with patients.’ (P 7)

‘Again, we are not recognised by our council.’ (P 14)

‘[…] management limitation is another thing, they only expect doctor to do ward rounds and is like we are forcing things […].’ (P 9)

## Discussion

Currently, the renal MDT in the Pietersburg hospital operates without pharmacists. The lack of their participation in renal the MDT is a historical problem pertaining to the classical role of the pharmacists. The role of pharmacists in the renal unit is currently limited to stock management and dispensing. This is in line with the results obtained from a recent study where majority of pharmacists were found to be more involved in the pharmaceutical product management such as ward stock inventory, compounding and dispensing (Katoue & Al-Taweel 2016; Sello & Dambisya 2014). While the integration of pharmacists in the wards to provide clinical services is common among First World countries such as the United States of America (USA) and and the United Kingdom (UK), it is still lacking in South Africa and other African countries (Bronkhorst et al. 2014; Gray et al. 2016; Mekonnen et al. 2013; Stemer & Lemmens-Gruber 2011).

The continuous absenteeism of pharmacists from the wards has created an environment where some of the participants in the renal unit believe that it is ‘fine working without’ pharmacists. This aspect may be related to the traditional practice of a pharmacy and lack of awareness and recognition of the role of pharmacists within the wards. Similarly, Krzyżaniak, Pawłowska and Bajorek (2018) found ignorance of pharmacists’ competencies and skills as one of the barriers. Therefore there is a need to raise awareness among healthcare professionals about the benefits of working with pharmacists in the wards (Alipour, Peiravian & Mehralian 2018). This should go along with the recognition of pharmacy services in the wards through collaborative efforts by all the stakeholders (health care professionals, healthcare institutions and their management, South African Pharmacy Council and the National and Provincial Department of Health). Employee recognition is an integral part of organisational management and is related to the basic needs of the employees (Brun & Dugas 2008). Hence pharmacists feel marginalised and discouraged to do ward rounds in the current set-up.

It is encouraging to note that although pharmacists are absent in the wards, the participants of the study believe that pharmacists can play a role in the management of renal patients. The inclusion of a pharmacist in the renal MDT can improve renal patients’ state of health and reduce drug-related problems (McNeely 2017; Salgado et al. 2014). Accordingly, participants indicated that pharmacists can review patients’ medication, work with prescribers during the prescribing stage, prevent the use of contraindicated drugs and to oversee dosage adjustments. Having a pharmacist undertaking these roles ensures effective use of medicines and improves the health outcome of patients and reduces the rate of readmissions (Shanika et al. 2018). Additionally, the involvement of a pharmacist in the early stages of drug therapy would serve as a source of patient customised pharmacological information to the team members. Given the current use of technology applications and abundance of information, pharmacists will play a major role in the dissemination and customisation of patient therapy.

Similar to a recent study (Katoue & Al-Taweel 2016), the participants in this study also highlighted patient education and counselling as one of the important roles to be played by pharmacists in the renal MDT. Patient education and counselling can reinforce patient adherence and understanding of medication. Therefore, there is a room to improve the renal patient care in the Pietersburg hospital by involving pharmacists in the provision of clinical services to the patients.

Results revealed a shortage of staff, pharmacists’ lack of clinical skills, negative attitude of pharmacists and other health care professionals and lack of recognition as the major barriers that limit the participation of pharmacists in the renal MDT. These findings are correlated because if the hospital does not have enough staffing, staff members will be overworked and have limited time to engage in professional development activities that will help them acquire more knowledge on therapeutics. The results in this study are compatible to those found by Ghazal and colleagues, as they reported lack of time, insufficient staffing and lack of motivation or vision of professional development to be the main barriers to the implementation of pharmaceutical care into pharmacies (Ghazal et al. 2014).

According to the South African Pharmacy Council (2018), pharmacists should be competent to participate as part of a healthcare team to ensure optimal pharmaceutical care. However, results from this study give the impression that undergraduate education may not be sufficient to ensure that pharmacists are competent to undertake this role. Similar results were also observed by Trinh et al. (2018) and Tegegn et al. (2018). James and Cole (2016) found that pharmacist interns in Sierra Leone were not prepared to practice in a MDT. There is a need to strengthen undergraduate pharmacy education in South Africa to be patient-orientated and empower pharmacists to practice in the MDT. Additionally, pharmacists should be life-long learners, striving towards continuous professional development and institutions of higher learning should develop relevant clinical short courses to support graduates of traditional product-orientated pharmacy curriculums. Even though clinical pharmacy practice in South Africa is not restricted to pharmacists with a postgraduate degree in clinical pharmacy, the lack of specialised clinical pharmacy postgraduates hampers the development and transition of pharmacy towards providing advanced direct patient clinical services (Sakeena, Bennett & McLachlan 2019; Trinh et al. 2018).

The discrepant attitude of some healthcare providers about the role of pharmacists being part of the MDT and patient care could be related to a lack of communication and/or coordination and lack of trust in the pharmacist’s abilities (Katoue et al. 2014). Interestingly, pharmacists believe that their roles are side-lined by doctors, but this could not be established from the doctors’ insights about having a pharmacist as part of the MDT in this study. This is in line with the results from a study conducted in Kuwait, on the role of pharmacists in parenteral nutrition therapy, as physicians were reported to assume total responsibility for the overall therapeutic decisions in collaborative teams (Katoue & Al-Taweel 2016).

Poor attitude of pharmacists to participate in MDTs may be explained by the lack of confidence and fear of new responsibilities among some pharmacists, which could have adversely affected their perception. In a recent study conducted by Rosenthal and colleagues, it was found that a culture exists among pharmacists to resist change, meaning pharmacists do not want to change from the traditional role of pharmacists to providing direct patient care (Rosenthal, Austin & Tsuyuki 2010).

## Recommendation and/or strategies to increase pharmacists’ participation in the renal multidisciplinary team

### Standardisation of the practice

To increase the participation of pharmacists in the MDT, it was recommended to standardise the procedure to include pharmacists as part of the MDT. In this context it would mean the development and implementation of pharmacy practice in the wards by including pharmacists as part of organisational ward structure recognised by the National and Provincial Departments of Health and hospital managements. This could be in the form of standard operating procedures, guidelines and job descriptions to guide the participation of pharmacists in the MDT. A standardised procedure for renal care within the MDT would improve patient safety and clinical appropriateness, enhance efficient resource utilisation, improve continuity of care and overcome any miscommunications (Katoue & Al-Taweel 2016). The results of this study are comparable to those found by Wagner and colleagues, where protocols and standardisation of procedures in late-stage CKD were reported to improve care and outcomes of the patients (Suleiman & Onaneye 2011).

### Skills development of both pharmacist and pharmacist assistants

There was an agreement among the participants about the need to foster pharmacists’ education on renal therapy.Therefore, participants recommended that workshops and short courses could be introduced to equip them with the necessary skills required. This is highly necessary taking into account that the majority of experienced pharmacists in hospitals may be graduates of the traditional product-orientated curriculum and for the purpose of continuous professional development of pharmacists in general. While short courses and workshops may provide an immediate solution for the current workforce, there is a need to reconsider the undergraduate pharmacy curriculum to embrace and support the acquisition and development of clinical skills through the inclusion of ward rounds during hospital experiential learning, use of case studies during lectures and tutorials, and role playing among other activities.

To overcome the issue of shortage of staff, participants stressed the need for the hospital to train more pharmacist assistants, which will reduce the workload while providing more time for pharmacists to move their services to the wards. The results were compatible with those found by Suleiman and colleagues, as education and hiring of more staff were found to improve the provision of pharmaceutical care (Suleiman & Onaneye 2011). Additionally, dispensing machines and technology can be used to reduce pharmacists’ dispensing workload and provide the necessary time to engage with patients in the wards.

### Recognition of pharmacist services within the wards

Employee recognition is an integral part of organisational management and is related to the basic needs of the employees (Brun & Dugas 2008). To raise the recognition of the pharmacy services, all stakeholders should be made aware of the role of a pharmacist within the MDT. Recognition of pharmacy practice and value will motivate and empower pharmacists to move their services to the wards. Furthermore, recognition of this practice would ensure availability of resources and ultimately improve patient care and outcome. This is in line with a study conducted by Sello and Dambisya (2014), that the service provided by pharmacists in the wards should be recognised and acknowledged by the Pharmacy Council and hospital management.

Pharmacist should note that recognition is built through high-quality pharmacist contributions (Snyder et al. 2010). Therefore, pharmacists would need to drive this recognition process through consistent ward rounds and provision of clinically useful recommendations and and this would ultimately stimulate the need for a pharmacist to be part of a team in the ward.

## Limitations of the research

The study was conducted at a tertiary hospital in Limpopo Province, and therefore may not apply to other hospitals offering renal care in South Africa and other countries. Additionally, the sample size was too small to generalise the findings from this study.

## Conclusion

A potential opportunity exists for pharmacists to offer direct renal care services within the renal MDT. Given the complex nature of pharmacotherapy in CKD patients, there is a need for better integration of pharmacists within the MDT. As a means to identify and gain pharmacists to become part of the MDT it was important and critical to point out perceptions of the barriers, and to encourage the expansion of the pharmacist’s role in the MDT. While pharmaceutical care services are limited at Pietersburg hospital, strategies were recommended to successfully implement and encourage the participation of pharmacists in the renal MDT, with the consideration of standardising the practice of pharmacists in the wards, equipping both pharmacists and pharmacist assistants with the necessary skills and recognise pharmacist services in the wards. More importantly, pharmacists have the potential to be frontiers in the drive to recognise pharmacists as essential team players within the MDT by continuously providing useful clinical recommendations. Further research is needed to evaluate the possibility of implementation and outcome of the proposed strategies.
